# Wolverines on the Move: A Multi‐Scale Analysis of Forest and Landscape Factors Influencing Wolverine Occurrence in Finland

**DOI:** 10.1002/ece3.71300

**Published:** 2025-04-21

**Authors:** Pinja‐Emilia Lämsä, Audrey Mercier, Andreas Lindén, Aarne Hovi, Miina Rautiainen

**Affiliations:** ^1^ School of Engineering Aalto University Espoo Finland; ^2^ Natural Resources Institute Finland (Luke) Helsinki Finland

**Keywords:** forest structure, multi‐scale analysis, remote sensing, species distribution, wildlife movement

## Abstract

Species distributions in forest‐dominated landscapes are closely tied to vegetation structure and heterogeneity, which can vary across spatial scales. As Fennoscandian wolverines recolonize their historical range in boreal forests, specific structural features linked to better resources, such as prey availability, cover, and suitable denning habitats, may promote occupancy in these areas. We studied wolverine ( *Gulo gulo* ) occurrence in mainland Finland between 2009–2010 and 2018–2022. We conducted a multi‐scale analysis using wildlife and field triangle data and Multi‐Source National Forest Inventory (MS‐NFI) remote sensing products. We applied generalized linear mixed models (GLMMs) to assess the influence of forest and landscape variables on the probability of occurrence at two spatial scales: local (3.13 km radius) and landscape (20 km radius). Occupied and unoccupied sites were distinguished by landscape fragmentation, tree volume, tree species composition, and distance to clearcuts. Sites were more likely to become occupied when forests were less fragmented and had broadleaved trees, while the probability of occurrence decreased if the total volume of trees was high or fresh clearcuts were in close proximity. Landscape scale seems to be more relevant than local scale when studying the overall forest structure's impact on wolverine occurrence. Our findings provide new insights into the occurrence of wolverines in Finnish boreal forests and could be used to aid species conservation and forest management planning.

## Graphical abstract

Using remote sensing products and wolverine snow‐track observations, we found that less fragmented forests with broadleaved trees promoted wolverine occurrence, while high total volume of trees and proximity to clearcuts hindered it. The landscape scale was more significant than the local scale in explaining the impact of forest structure on occurrence. Our results offer new insights on the occurrence of wolverines in the boreal forests.

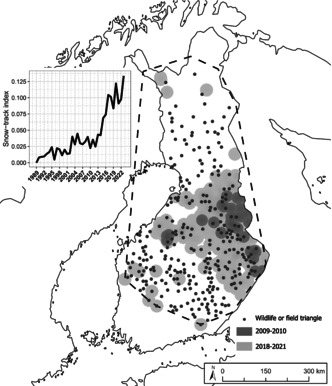

## SUOMENKIELINEN TIIVISTELMÄ

Lajien levinneisyys kytkeytyy metsävaltaisilla alueilla kasvillisuuden rakenteeseen ja koostumukseen, jotka voivat vaihdella spatiaalisen skaalan mukaan. Fennoskandian ahmat tekevät paluuta historiallisille elinalueilleen boreaalisiin metsiin. Monipuolisia resursseja tukevat metsien ja maiseman rakenneominaisuudet voivat kannustaa tietyn alueen asuttamiseen. Tutkimme ahman ( *Gulo gulo* ) esiintymisen muutoksia Manner‐Suomessa vuosien 2009–2010 ja 2018–2022 välillä. Aineistona käytimme riista‐ ja peltokolmioaineistoa sekä monilähteisen valtakunnan metsien inventoinnin (MVMI) kaukokartoitustuotteita. Yleistettyjen lineaaristen sekamallien (generalized linear mixed models) avulla analysoimme metsän ja maiseman rakenteiden vaikutuksia esiintymistodennäköisyyteen kahdella eri spatiaalisella skaalalla: paikallisella tasolla (3.12 km säde) ja maisematasolla (20 km säde). Asutut ja asuttamattomat alueet erosivat maiseman pirstoutuneisuuden, puuston kokonaistilavuuden, puulajikoostumuksen sekä avohakkuiden etäisyyden osalta. Ahmojen esiintymistodennäköisyys kasvoi, kun metsät olivat vähemmän pirstoutuneita ja lehtipuuvaltaisempia. Suurempi puuston kokonaistilavuus ja läheinen etäisyys avohakkuisiin alensivat esiintymistodennäköisyyttä. Tulosten perusteella maisemataso on paikallista merkityksellisempi, kun tutkitaan metsien yleisen rakenteen vaikutusta ahmojen esiintymisen todennäköisyyteen. Tutkimustulokset lisäävät ymmärrystä ahmojen esiintymisestä boreaalisissa metsissä.

## 1 Introduction

In forest‐dominated landscapes, species distribution largely links to the structure of vegetation and its heterogeneity through resource provision (Davies and Asner 2014). The effect of these structures and compositions, however, may vary across different spatial scales (Stuber and Fontaine 2019). As global biodiversity declines (Isbell et al. 2023), understanding the importance of vegetation composition and structure across spatial scales becomes increasingly important for assessing species management and to sustain ecosystem functioning (Davies and Asner 2014; Jackson and Fahrig 2015).

The structure of Finnish forests has changed notably during the last decades due to forestry practices. Despite the increase in total forest area and tree growth (Korhonen et al. 2024), the general biodiversity in forests has declined and old‐growth forest areas have become scarcer (Mönkkönen et al. 2022). Landscape connectivity is an essential component of functional ecological networks, and thereby significant for maintaining viable populations (Baguette et al. 2013). Moreover, it facilitates animal dispersal, which is influenced by species' movement dynamics and their interactions with changing environments.

The wolverine ( *Gulo gulo* ) inhabits remote areas of tundra, alpine, and taiga habitats across the Northern Hemisphere (Macdonald et al. 2017). Over two‐thirds of Finnish wolverines inhabit boreal forests, while the rest occupy regions with treeless oroarctic vegetation in the reindeer husbandry area (Kojola et al. 2023). As Fennoscandian wolverines are recolonizing their historical range after gradually receiving protection during 1973–1982 (Moqanaki et al. 2023), they have been dispersing from central eastern Finland (Kainuu region) towards south and southwest Finland (Kojola et al. 2023), and the population size in Finland has shown an approximately tenfold increase during 1989–2023 (Kojola et al. 2023). Nevertheless, the wolverine is listed as endangered in Finland (Hyvärinen et al. 2019), and therefore meets the criteria for conservation measures. The Fennoscandian wolverine population remains threatened due to small population size, low genetic viability, and hence low effective population size, poaching, and fragmented distribution (Lansink et al. 2022). Previous studies highlight that Fennoscandian wolverine habitats should be protected from intensive anthropogenic development and the priority should be on maintaining and restoring connectivity of high‐quality habitats to maintain gene flow between populations (Bujnáková et al. 2024; Koskela et al. 2013a; Lansink et al. 2022). The protection established in the late 1980s coupled with the subsequent increase in population size offers an excellent opportunity to study the factors influencing wolverine occurrence in Finland.

In Finland, 86% of the total land area (304,000 km^2^) is classified as forestry land. Of this 262,000 km^2^, 67% is production forest and an additional 10% is partly available for timber production (Korhonen et al. 2024). Although wolverines occupy habitats with varying vegetation types (Larivière and Jennings 2009), forestry practices influence the habitats of the Finnish wolverine population as they mainly inhabit the extensive network of boreal forests and peatlands within (Kojola et al. 2023). Given that the differences between different forest types and peatlands relate to vegetation structure, examining the effects of forest and landscape structure could aid species conservation and management planning. This is particularly the case in countries like Finland, where recommendations for forestry practices can have broad, nationwide implications.

Remote sensing provides a way to assess habitat characteristics across large areas such as entire countries (Gough and Rushton 2000), which assists when studying elusive large carnivore species with vast home ranges. Despite its evident advantages, thus far, remote sensing has not been extensively employed when investigating wolverines or mustelids in general. Previous remote sensing research on wolverine habitats has mainly focused on detecting snow cover and its changes in the Nearctic region (e.g., Barsugli et al. 2020; Copeland et al. 2010; McKelvey et al. 2011). Although the habitat selection of wolverines has been previously explored in Fennoscandia (Fisher et al. 2022), little is known of the types of habitats utilized during range expansion.

In this paper, we investigate the effect of forest and landscape structures on wolverine occurrence across two spatial scales. We hypothesize that wolverines occupy new areas characterized by structures associated with old‐growth forests, a more continuous landscape further from clearcuts, a higher proportion of conifers, and a lower proportion of broadleaved trees and pine. Our rationale for this hypothesis is that according to a study in one Finnish province, wolverines favor mires and remote, mature forests (particularly mixed or coniferous), while they tend to avoid young deciduous forests and settlements (Koskela et al. 2013a). Furthermore, wolverines favor a higher forest cover but show no additional association with canopy cover (Malcangi et al. 2024). As low‐elevation forested habitats in Finland often lack preferred den characteristics such as boulders and steep terrain (Fisher et al. 2022), understory vegetation and decaying wood matter might become more important for reproduction, especially in areas without persistent spring snow (Jokinen et al. 2019). Less‐fertile sites that often have a higher volume of pine would more likely lack these features. While fresh clearcuts might provide scavenging opportunities, as field voles ( *Microtus agrestis* ) become more abundant (Savola et al. 2013), we hypothesize they are avoided due to increased human disturbance and highly changed forest structure. Throughout its distribution, the species is affected by human disturbance (Fisher et al. 2022), which tends to increase in areas fragmented by anthropogenic land‐use changes (Haddad et al. 2015).

Our research questions are: (i) Which forest and landscape structures explain the wolverine occurrence? (ii) Do the structures that explain wolverine occurrence differ when analyzed at different spatial scales? (iii) What is the most appropriate spatial scale for studying wolverine occurrence? The study focuses on the entire mainland Finland and utilizes remote sensing‐based products on forest and landscape structure.

## 2 Materials and Methods

### 2.1 Wolverine Location Data

The locations of wolverine snow‐track observations were obtained from the winter wildlife triangle data, a nationwide monitoring program, which is coordinated by the Natural Resources Institute Finland (LUKE). The wildlife‐ and field triangle data contain annual information about wildlife abundance and its changes. Wildlife triangles are 12 km line transects, shaped like equilateral triangles with 4 km long sides, located in forest landscapes and surveyed since 1989. Field triangles, on the other hand, are 6 km triangle‐shaped line transects (2 km per side) that have been surveyed since 1999 (Lindén et al. 1996; LUKE 2024). Field triangles are located in small‐scale agricultural mosaic landscapes with about half of the census being in agricultural fields and the rest in forests, yards, and built environments (LUKE 2024). Nationwide, over 2000 triangles cover both forest and agricultural environments, with around 1000 surveyed annually. During the winter, wildlife and field triangles (hereafter both referred to as “triangles”) are typically surveyed by skiing along the census line, by one or several volunteers, marking on a map the observed snow‐tracks of game animals crossing the survey line. The winter survey in wildlife triangles is done between the January 15 and February 29 in most parts of Finland, but in the north, the survey period is extended until the 15th of March. Field triangles are surveyed between the January 1 and March 5.

**FIGURE 1:**
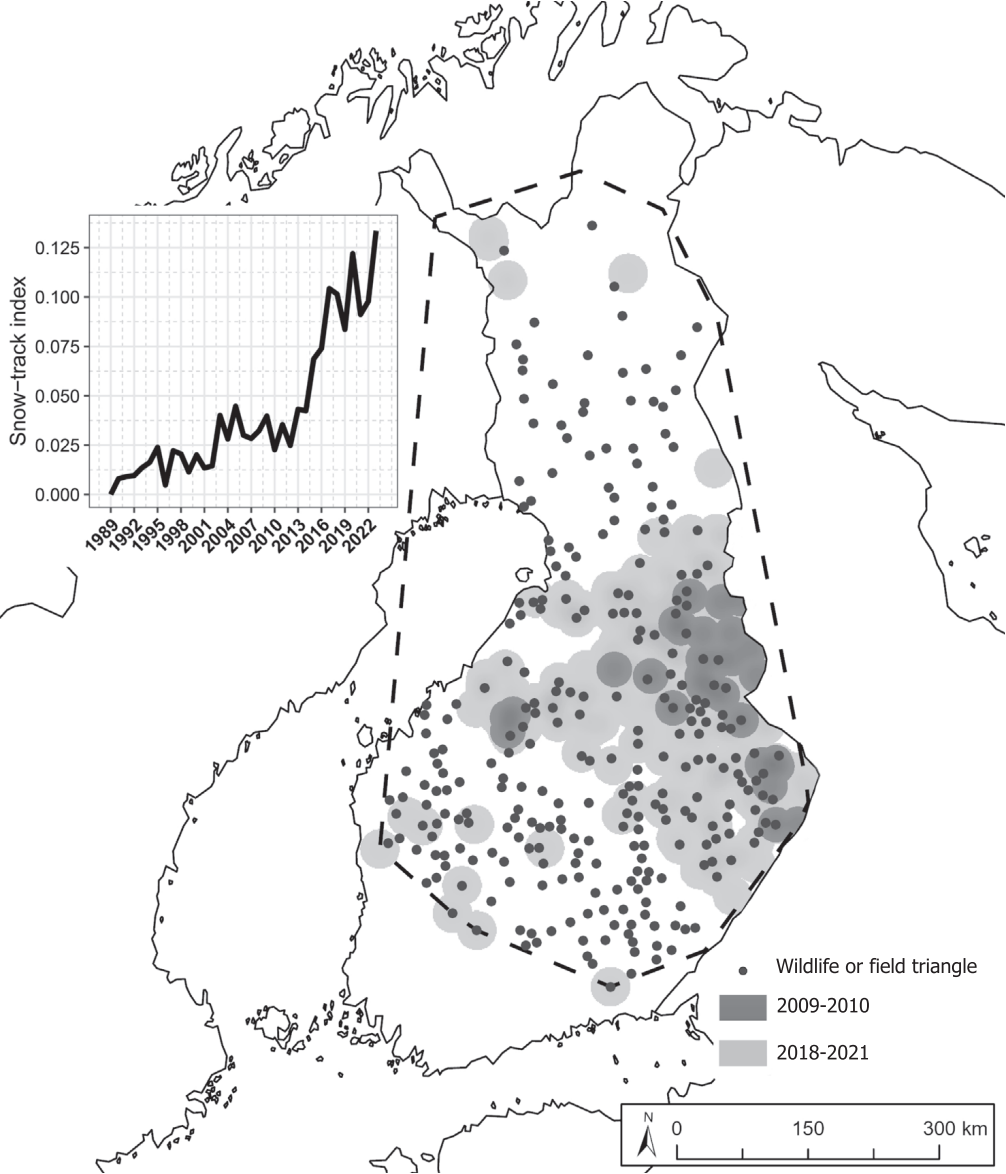
Wolverine distribution in Finland (land area 304,000 km^2^), based on wildlife and field triangle data, divided into two periods. Dark gray illustrates areas occupied by wolverine in the first period (2009–2010) and light gray in the second period (2018–2021). Black dots (•) represent the wildlife and field triangles selected for the study. The dashed black line represents 100% the minimum convex polygon used to estimate wolverine range. The plot shows an increase in wolverine abundance from 1989 to 2023, based on the snow‐track index (number of tracks/10 km/day). The index measures frequencies of wolverine tracks, and with some reservation, the relative population size (Lindén et al. 1996).

The study area is located in mainland Finland and consists of an area inside a 100% Minimum Convex Polygon (MCP), in which wolverines have been observed during 2009–2023 (Figure 1). MCP estimates the species' range by drawing a convex polygon around the selected observations (Hayne 1949). Compared to the mountainous terrain of wolverine habitats in Scandinavia, the Finnish landscape is characterized by relatively flat topography and extensive boreal forests. Wolverine occurrence (variation in the distribution of wolverines) is studied between two distinct time frames: 2009–2010 (hereafter referred to as “the first period”) and 2018–2021 (hereafter referred to as “the second period”). The former was chosen for being the earliest period with available Finnish Multi‐Source National Forest Inventory Raster Maps (MS‐NFI) (Tomppo et al. 2014), while the latter was chosen as it describes the current status of Finnish wolverine population and corresponds to the most recent MS‐NFI products. These time frames align with the typical lifespan of wolverines, thereby enabling meaningful examination of population changes (Andrén 2018). To account for variation between different individuals and effectively identify important structural features, a larger sample size is needed (Baguette et al. 2013). Therefore, we included observations from 4 years (2018–2021).

The workflow for selecting triangles for the analysis was the following: (1) triangles that had no wolverine observations in 2009–2010 were selected, (2) from those, the ones that were inside a 100% MCP were selected to ensure that the selection corresponds to the species' range, (3) triangles within a 20 km distance of the country border were excluded because data from neighboring countries (e.g., Russia) is unavailable, and finally, (4) a random selection of triangles no closer than 10 km from each other was made to avoid exceeding overlap between triangle buffers at larger spatial scales. Lastly, to reduce potential bias, we excluded triangles with more than nine snow‐track accumulation days, as tracks become harder to detect over time, particularly in open areas. Accumulation days indicate the number of days tracks have been accumulating since the last snowfall or the preliminary snow‐track mapping.

### 2.2 Forest and Landscape Structures

Forestry practices vary significantly between countries, influenced by local policies, ecological conditions, and ownership structures. According to the Finnish Statistical Yearbook of Forestry (Kulju et al. 2023), over half of Finland's forests are privately owned, with an average compartment size of 1–2 ha. The typical rotation period for forest stands ranges from 80 to 120 years, influenced by the latitude of the location. The dominant tree species is scots pine ( *Pinus sylvestris* ), followed by Norway spruce ( *Picea abies* ), silver birch ( *Betula pendula* ), and downy birch ( *Betula pubescens* ). At the country level, coniferous trees are harvested at a higher rate than broadleaved species due to their greater abundance. However, at the stand level, broadleaved species are often harvested at a younger age than conifers. Approximately one‐third of Finland consists of peatlands, about half of which have been drained and are used for forestry. Among forest regeneration techniques, clearcutting is the most commonly used method.

MS‐NFI remote sensing products from LUKE were employed to describe structural features of forests and to calculate landscape metrics. They provide nationwide forest resource estimates from satellite images, field measurements, and other geospatial data (Tomppo et al. 2008). MS‐NFI products are not available for all years; hence, the year 2011 was chosen to represent the forests during the first period and products from the year 2021 to represent the second period.

The satellite imagery used included Landsat 5 TM and Landsat 7 ETM+ imagery from years 2009–2011, and Sentinel‐2A MSI, Sentinel‐2B MSI, and Landsat 8 OLI from the year 2021 (Tomppo et al. 2014). The product from the year 2011 has a spatial resolution of 20 × 20 m and from the year 2021 a spatial resolution of 16 × 16 m (Tomppo et al. 2014). The products are described in detail in Tomppo et al. (2014).

A set of environmental covariates, describing structural features of forests and landscape, was defined (see Section 2.3). Building on previous research on wolverine ecology and habitat preferences in Finland (Koskela et al. 2013a; Malcangi et al. 2024), and the known avoidance of human disturbance (Fisher et al. 2022), we identified a set of potentially relevant explanatory variables. These included forest attributes describing species composition (volume of different tree species, percentage of different tree species), tree and forest structure (stand basal area, standing mean height, stem diameter, stand age, total volume of trees), canopy cover and site main type (proportions of mineral soil, pine‐dominated mire, spruce‐dominated mire, and open peatlands). Additionally, we incorporated landscape characteristics, including fragmentation (patch density, edge density), and distances to key features (anthropogenic landscape, roads, clearcuts and water bodies). These covariates (Table A1) were collected for both time frames and two spatial scales by using “landscapemetrics” (Hesselbarth et al. 2019) and “terra” packages (Hijmans 2024) from the statistical programming environment R, version 4.4.0 (R Core Team 2024).

**FIGURE A1:**
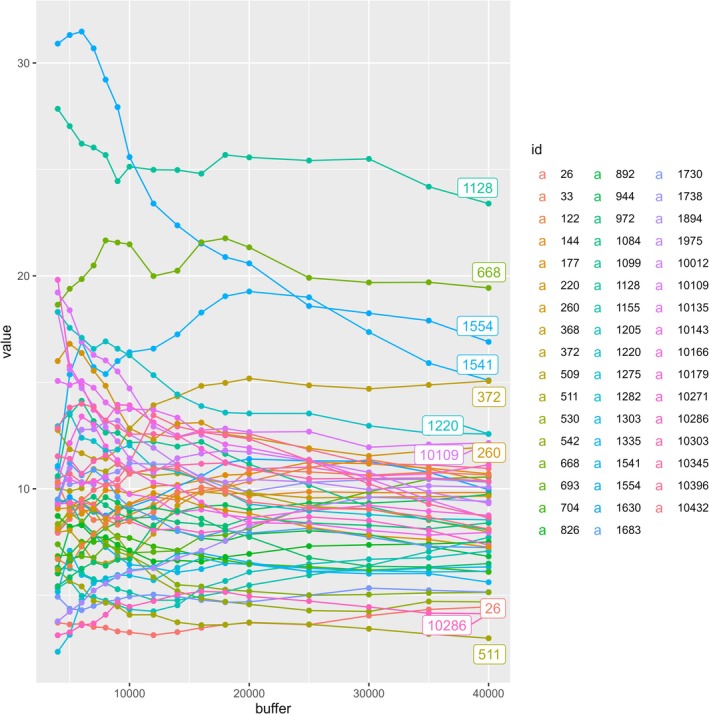
Plot of patch density values across buffer distances, differentiated by “Triangle ID”. Variability of patch density stabilizes after 20 km buffer distance. Based on these findings, a scale of 20 km was selected as the optimal scale for further analysis as plot suggested consistency in patch density across larger scales.

Two spatial scales corresponding to circular windows around the centroids of the sampled triangles with buffer radii of 3.13 and 20 km were applied. The 3.13 km buffer radius provides representation of a local scale. Although wolverines are generally mobile animals, females often restrict their movements to a small local area during the denning season. Based on a recent study in Sweden, during the early denning season, wolverine females travel only short distances (max 2.1 km until mid‐April) between den sites and spend very little time outside the denning area (Aronsson et al. 2023). However, as such distance does not cover the entire triangle, we increased the buffer size to exceed 1 km beyond triangle vertices. The 3.13 km buffer radius accurately captures the area around each triangle, as well as the limited range used by females during the early denning season. The 20 km radius represents landscape scale, which was chosen based on wolverine dispersal distance in the Scandinavian mountain areas (51–60 km, Vangen et al. 2001) and preliminary analyses (Figure A1). As the landscape metrics presented low variability beyond 20 km, the landscape scale buffer size was applied as 20 km instead of 51–60 km. Moreover, selecting a 20 km buffer size allowed for a larger number of triangles in the analysis compared to buffer sizes of 51–60 km, as minimizing overlap between buffer areas was necessary to ensure the independence of triangles.

### 2.3 Data Analysis

Data exploration was implemented following the protocol described in Zuur et al. (2010), which included checking for outliers, assessing independence of observations, evaluating collinearity among predictors, and assessing relationships between variables. The goal of the data exploration was to identify data characteristics and potential problems. As a result, one outlier triangle from Western Lapland was removed from further analysis, as it had a very different landscape structure compared to the rest of the triangles.

Possible issues with collinearity of covariates were assessed using Pearson's correlation coefficients and variance inflation factors (VIF). Environmental variables measured in both 2011 and 2021 were averaged if they showed high positive Pearson's correlation coefficients (*r* > 0.7). Next, a VIF analysis was conducted to reduce dimensionality. The procedure involved pre‐selecting five covariates based on their theoretical or practical importance, and their Pearson's correlation coefficients were checked to ensure that the absolute value of the correlation did not exceed 0.7, thus avoiding strong correlations. These five covariates were retained in the analysis regardless of their VIF values. The set of covariates was chosen to represent forests and landscapes from versatile aspects: volume of pine (relates to general site fertility, portraying less‐fertile areas), total volume of trees (a summative characterization of forest structure as it relates to structural features like tree height, tree diameter and number of trees), percentage of broadleaved trees (a proxy for characterizing biodiversity), distance to clearcuts (a common element in Finnish boreal forests), patch density (a traditional metric for describing measure of fragmentation). As for non‐preselected covariates, variables with the highest VIF value were iteratively removed until all remaining variables were under a threshold (VIF < 3). Based on the VIF analysis, only the preselected covariates, which had VIF values below 3, were retained (Table 1; Table A2).

**TABLE 1:** Forest and landscape variables included as covariates in the generalized linear mixed models (GLMMs). Covariates preselected for VIF analysis are presented with an asterisk (*).

Variable	Short name	Description	Unit
Volume of pine*	VolPine	Average volume (growing stock) of pine within a circular window. Related to general site fertility. Raster from MS‐NFI.	m^3^
Total volume of trees*	VolTotal	Average total volume (growing stock) of trees within a circular window. Related to tree height, tree diameter, and number of trees. Raster from MS‐NFI.	m^3^
Proportion of broadleaved trees*	Broadleaved	Average percentage of broadleaved trees within a circular window. Related to site biodiversity. Volume of broadleaved trees divided by the total volume of trees. Raster from MS‐NFI.	%
Distance to clearcuts, 2009–2010*	Clearcut1	Average distance to clearcuts in the first period. Clearcut was defined as the MS‐NFI “*forest land*” pixels that do not fulfill the forest definition (canopy cover > 10%, tree height > 5 m) and were subtracted from unfiltered “*forest land*” raster.	m
Distance to clearcuts, 2018–2021*	Clearcut2	Average distance to clearcuts in the second period. Clearcut was defined as the MS‐NFI “*forest land*” pixels that do not fulfill the forest definition (canopy cover > 10%, tree height > 5 m) and were subtracted from unfiltered “*forest land*” raster.	m
Patch density*	PatchD	Forest fragmentation metric calculated from MS‐NFI “*forest land*” raster filtered based on forest definition (canopy cover > 10%, tree height > 5 m).	Number per 100 ha

To analyze the effects of the forest and landscape structures on the probability of occurrence in the second period at the triangles where wolverines were not observed in the first period (hereafter referred to as “probability of occurrence”), Generalized Linear Mixed Models (GLMMs) were applied with a binomial error distribution, and a logistic link‐function. The binary response variable was presence (‘1’) or absence (‘0’) of wolverines in an observation *i* in a triangle *j*. Grouping variable “Triangle ID” was included as the random intercept, that implies a correlation structure between observations from the same triangle, as each triangle was counted multiple times over different years. Year was not included as a random effect since its estimated standard deviation was zero. Log‐transformed *effort* (counted census line in 10 km × accumulation time in days) was added to all models as a covariate to account for sampling effort. All fixed effects covariates presented in Table 1 were continuous, and we chose to standardize them to zero mean and unit variance to facilitate comparison of effects between covariates.

The degree of spatial autocorrelation was evaluated for the most parsimonious model by estimating the Moran's I with the “spdep” R package (Bivand 2022). Estimations were calculated for both scales based on the random effect estimates associated with the “Triangle ID” as this assesses spatial dependency captured within the random effects. The results suggested that there was no significant spatial autocorrelation (local scale *p* = 0.202, landscape scale *p* = 0.088), and therefore no additional structures for spatial dependence were included in the model.

GLMMs were fitted using the function “glmmTMB” from the R package “glmmTMB” (Brooks et al. 2017). Separate models were fitted for covariates on the local (3.13 km) and landscape (20 km) scales. Akaike information criterion corrected for small sample sizes (AICc, Burnham and Anderson 2002) was used for model selection. All possible combinations of the covariates were fitted for both scales, totaling 64 models per spatial scale. The models were fitted with maximum likelihood and then compared using AIC_c_ (Table A3). Model assumptions were verified by plotting residuals against fitted values, against each covariate, and against the random effects of “Triangle ID”. These were plotted with “DHARMa” R package (Hartig et al. 2022).

The goodness‐of‐the‐fit of the best models was assessed using: the area under the curve of a ROC plot (AUC), true skill statistics (TSS), and a Brier score. First, predicted probability values were transformed into observed absences (0) and presences (1), based on the optimal threshold for binary classification, which was calculated with the “optCutoff” function from the “Mkclass” R package (Kohl 2023). Then, a confusion matrix was calculated with the “caret” R package (Kuhn 2008). The predictive accuracy of the most parsimonious models was measured by AUC using the “PresenceAbsence” R package (Freeman and Moisen 2008), TSS, and Brier score using the “DescTools” R package (Signorell et al. 2024). An AUC of 0.5 indicates that the model is no better than by chance, 0.7–0.8 suggests the model to be acceptable, 0.8–0.9 is considered excellent, and over 0.9 would be outstanding (Hosmer et al. 2013). TSS ranges from −1 to +1, where −1 would indicate unsuitable model fit, a value 0 indicates model performance is no better than random, and 1 that the model is suitable (Allouche et al. 2006). The Brier score measures the accuracy of probabilistic predictions in a similar manner as R^2^, but inversely. The score ranges from 0 to 1, with 0 indicating a perfect prediction (Fenlon et al. 2018).

## 3 Results

Altogether, our data consisted of 884 triangle surveys representing 279 unique triangles, of which 269 were wildlife triangles and 10 were field triangles. In total, 13.12% of the triangles had wolverine tracks. At the local scale, a set of competing models (nine models with ΔAIC_c_ < 2, Table A3) was found to best explain the variation of the probability of occurrence in the second period. The most parsimonious model included the patch density and the percentage of broadleaved trees. As this model was nested within all other competing models, we can interpret the most parsimonious model as the single best one, and distance to clearcuts in the first period, distance to clearcuts in the second period, total volume of trees, and volume of pine as uninformative covariates (Arnold 2010).

At the landscape scale, the probability of occurrence was best explained by two competing models (ΔAICc < 2, Table A4). The covariates included in the best model were distance to clearcuts in the first period, distance to clearcuts in the second period, patch density, total volume of trees, and percentage of broadleaved trees. The second‐best model had only one additional parameter (volume of pine) compared to its competitor. Thus, the volume of pine can be interpreted as an uninformative covariate, and the most parsimonious model (Table A4) can be considered the single best one (Arnold 2010).

Model validation revealed no major violations of model assumptions. Metrics of accuracy were calculated for the most parsimonious local scale model and the most parsimonious landscape scale model. Based on AUC, model performance was acceptable, TSS indicated models to be suitable, and Brier score indicated predictions to be accurate in both models (Table 2). The random effects of “Triangle ID” had a standard deviation of 2.129 for local scale and 1.894 for landscape scale, indicating variability in wolverine occurrence that could not be explained by the fixed effects (Table 3).

**TABLE 2:** Metrics of accuracy for wolverine generalized linear mixed models (GLMMs) at two different spatial scales.

Model	AUC	TSS	Brier Score
Local scale	0.710	0.334	0.118
Landscape scale	0.768	0.447	0.112

*Note:* Metrics are calculated based on the fixed effects.

Abbreviations: AUC, area under the ROC (receiver operating characteristic) curve; TSS, true skill statistics.

**TABLE 3:** Standardized coefficients of the most parsimonious logit‐link binomial mixed model at the local and the landscape scale.

Variable	Estimate	SE	*Z*‐value	*p*‐value
Local scale				
*α* Intercept	−3.404	0.399	−8.535	< 0.001
***β***_***1***_ **Broadleaved**	**0.663**	**0.212**	**3.134**	**0.002**
***β***_***2***_ **PatchD**	**−0.994**	**0.298**	**−3.334**	**< 0.001**
*β*_*3*_ Effort	0.068	0.151	0.448	0.654
*a*_*j*_ among‐site SD	2.156	—	—	—
Landscape scale				
*α* Intercept	−3.499	0.376	−9.304	< 0.001
***β***_***1***_ **Broadleaved**	**0.893**	**0.241**	**3.709**	**< 0.001**
***β***_***2***_ **VolTotal**	**−0.687**	**0.243**	**−2.827**	**0.005**
***β***_***3***_ **Clearcut1**	**−4.181**	**1.214**	**−3.445**	**< 0.001**
***β***_***4***_ **Clearcut2**	**2.786**	**0.881**	**3.163**	**0.002**
***β***_***5***_ **PatchD**	**−0.842**	**0.240**	**−3.511**	**< 0.001**
*β*_*6*_ Effort	0.062	0.148	0.419	0.675
*a*_*j*_ among‐site SD	1.894	—	—	—

*Note:* Variables with statistically significant effects are **bolded**.

**FIGURE 2:**
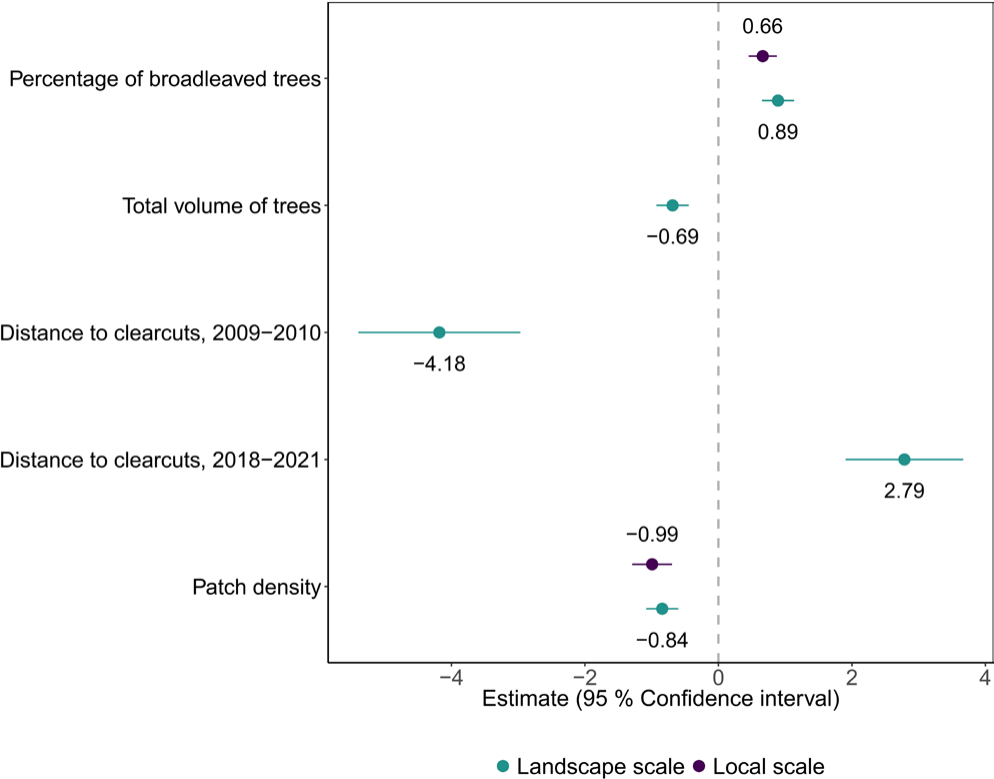
Estimated standardized coefficients (β ± 95% confidence interval) illustrating effect of each standardized forest and landscape covariate on wolverine occurrence at local and landscape scales.

**FIGURE 3:**
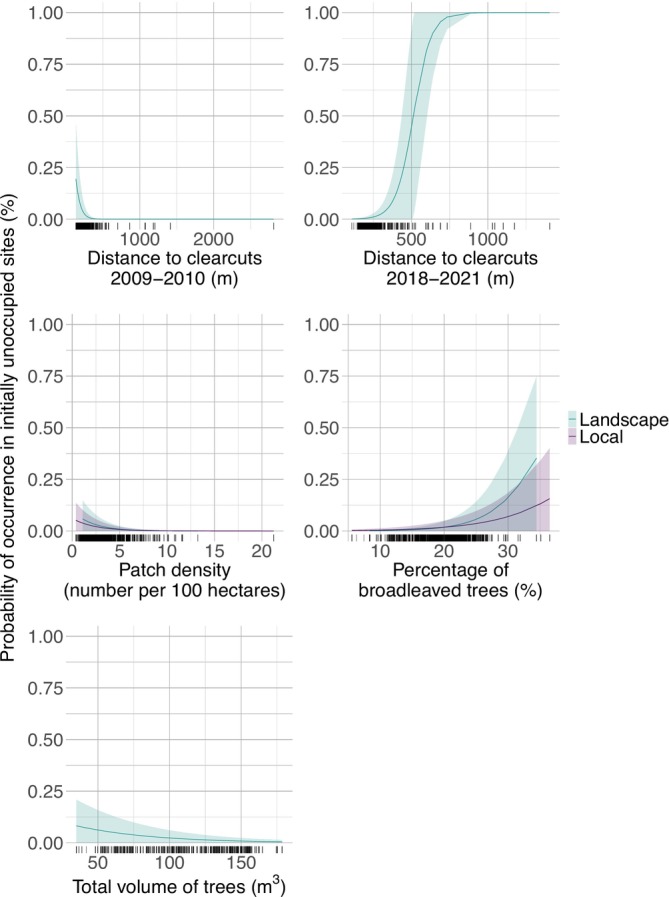
Predicted probability of occurrence in initially unoccupied sites based on each environmental covariate at the local and landscape scales. Shaded areas present 95% confidence intervals. Black tick marks along the *x*‐axis illustrate the distribution of the covariates.

The most parsimonious local scale model had two statistically significant variables explaining the probability of occurrence (Table 3, Figures 2 and 3). The probability was negatively related to the patch density and positively affected by the percentage of broadleaved trees. At the landscape scale, the most parsimonious model had five statistically significant variables explaining the probability of wolverine occurrence (Table 3, Figures 2 and 3). The probability was negatively affected by the distance to clearcuts in the first period, while the distance to clearcuts in the second period had a positive effect on it. The patch density and the total volume of trees were negatively related to the probability of occurrence, whereas the percentage of broadleaved trees had a positive relation.

The effect of the percentage of broadleaved trees and the total volume of trees was slightly larger at the landscape scale compared to the local scale (Figures 2 and 3). The distance to clearcuts in the first period and the distance to clearcuts in the second period were not significant at the local scale, while they had significant and opposite effects at the landscape scale (Figures 2 and 3).

## 4 Discussion

Our findings imply that wolverine occurrence was best explained by the distance to clearcuts, as well as to a smaller extent, by the tree species composition, tree volume, and landscape fragmentation. Generally, in occupied sites, forests were less fragmented and tended to be dominated by broadleaved trees, total volume of trees was moderate, and the sites were not in close proximity to fresh clearcuts.

In alignment with our hypotheses, wolverines preferred to occupy areas with smaller patch density and, therefore, a more continuous landscape. Landscape characteristics such as patch density and connectivity influence the effects of anthropogenic landscape fragmentation that can contribute to increased disturbance, reduced habitat availability, and obstructing dispersal and gene flow (Haddad et al. 2015; Sawaya et al. 2019). Larger forest patches are likely further away from human development and infrastructure such as roads and settlements, making them less accessible for humans and therefore providing habitats with less human disturbance. Mountainous protected areas provide low‐disturbance refuges for wolverines (Fisher et al. 2022). As mountainous areas are lacking in Finland, such refuges could be found in continuous forest patches that are harder to access by humans.

Deviating from our hypotheses and prior research (Koskela et al. 2013a), wolverines occupied areas with a higher proportion of broadleaved trees. Forests with a higher percentage of broadleaved trees support a wide range of species, offering a structurally complex environment with various ecological niches (Thuiller et al. 2006). In boreal forests, understory biodiversity benefits from broadleaved trees when stand density is modest (Salemaa et al. 2023). Areas with higher biodiversity could offer a greater abundance of prey animals and cover for foraging and protection (Castagneyrol and Jactel 2012), thus increasing the likelihood of wolverine occurrence. In the forested areas of Finland, the primary prey species for breeding female wolverines include mountain hares, grouse, small rodents, and moose (Koskela et al. 2013b). Vegetation structure, especially understory cover and canopy cover, is an important factor for mountain hares (Hiltunen et al. 2004; Malcangi et al. 2024), grouse (Mazziotta et al. 2024), and voles (Savola et al. 2013). Moose prefer forests with heterogenous vegetation, and both moose and field voles are known to use forests in younger succession stages (Melin et al. 2013; Savola et al. 2013). Furthermore, denser cover of deciduous trees might be favored during denning season, as was detected for one denning female in a Canadian study (Jokinen et al. 2019). Moreover, as wolverines favor wolf presence for scavenging opportunities based on a study in eastern Finland (Koskela et al. 2013a), forests with higher biodiversity and heterogeneous structure could provide cover from intraguild predation.

Prior studies suggest that wolverines favor higher forest cover and mature forests in Finland (Koskela et al. 2013a; Malcangi et al. 2024). However, our results imply that wolverines are unlikely to occupy areas with high total volume of trees at the landscape scale. Higher volume of trees could indicate denser forest cover and an older age class. Nevertheless, the proportion of mature forests in southern Finland is low and they tend to be more fragmented (Korhonen et al. 2021). Consequently, favorable structures might become too isolated and scarce for effective range expansion. Accordingly, our results imply that wolverines prefer areas with less fragmented forested landscape. On the other hand, in accordance with Koskela et al. (2013a), wolverines favoring moderate‐to‐low total tree volume could indicate preference for mires or treeless peatlands. Close proximity to peatlands supports food caching behavior (van der Veen et al. 2020). As snow depth continues to decrease in Finland due to climate change (Luomaranta et al. 2019), peat formations may serve as alternative food storage sites for wolverines when snow caching becomes impracticable.

Corroborating our hypotheses, wolverines were less likely to occupy areas if fresh clearcuts were in close proximity. In contrast, areas that had fresh clearcuts in the 1st period were more likely to be occupied. Avoidance of recent clearcuts or active clearcutting sites may be attributed to avoiding human activity (Barrueto et al. 2022). Ten‐year‐old clearcuts often have a denser shrub layer with a mixture of deciduous and coniferous species, and they tend to lack disturbing human activities, thus making them more favorable than recent logging sites. Logging areas, especially their edges, might provide foraging opportunities and paths for wolverines; meanwhile, the interiors of logging sites are usually avoided (Scrafford et al. 2017). However, wolverines occasionally take the risk of using active harvest areas for foraging opportunities (Scrafford et al. 2017). Nevertheless, clearcuts increase patch density, which is an unfavorable landscape structure based on our results.

Our results suggest that forest structure is more relevant on the landscape scale than on the local scale for understanding wolverine occurrence in boreal forests. However, the processes investigated in this study are forest‐related, and other factors not included could have an effect on the local scale (e.g., human infrastructure or factors related to peatlands). Ecological processes operate at different spatial scales (Zelnik et al. 2024), and for wolverines, broader landscape‐level habitat structures and resources could influence the occurrence more effectively than local habitat. As wolverines have relatively large home ranges (Persson et al. 2010), landscape scale could better capture larger spatial needs, while local scale might miss some broader habitat features related to forest structure. Moreover, the Finnish forest landscape is a mosaic of predominantly small privately owned forest patches. The landscape scale could better capture the structural features of forests and landscapes that support sufficient home range sizes for wolverines.

In expanding wolf populations, individuals settle first in the highest‐quality habitats (Planillo et al. 2024). As population density increases, later dispersers are pushed into progressively lower‐quality areas, facing fewer resources and greater human disturbance. Therefore, if wolverines follow a similar pattern, occupying higher‐quality habitats first, our findings may reflect characteristics of those habitats. Based on our results, forest and landscape structures could play a significant role in shaping wolverine distribution in Finland. While adaptable to varying habitats (Lofroth and Krebs 2007; Macdonald et al. 2017), wolverines often prefer higher‐elevation environments (Copeland et al. 2010) with steep and rugged terrain (May et al. 2012; Poley et al. 2018) and taluses (Carroll et al. 2021). Steep high‐elevation terrain oftentimes lacks human disturbance caused by anthropogenic development, which could partly explain the preference (Fisher et al. 2013). Due to Finland's predominantly lowland terrain, it lacks the steep landscapes that can be found in regions such as the Scandinavian mountain areas. In lowland areas, wolverines may rely more on forest and vegetation structure rather than the topographical features associated with high elevations. A study in northern Alberta (Canada) showed two female wolverines denning under partially uplifted root masses of trees and under decayed logging debris (Jokinen et al. 2019). The favoring of certain structural features of vegetation could reflect their role in providing resources such as prey animals, denning sites, and cover from intraguild predation. Furthermore, areas with favored structures, such as 10‐year‐old clearcut sites, may coincide with lower human activity and thus increase suitability. Nevertheless, in Finland, forestry practices have broad influences on species, their habitats, and general biodiversity (Mönkkönen et al. 2022).

Although expanding their range in Finland, wolverines are facing critical conservation challenges globally. Climate and landscape change, declining habitat connectivity, and human‐caused mortality (including fur harvest, predator control, and poaching) pose major threats to the species throughout its range (Fisher et al. 2022). Especially human disturbance, anthropogenic mortality, and climate change threaten the wolverine population in North America (Day et al. 2024; Scrafford et al. 2024; Fisher et al. 2022), and wolverine has been listed as threatened in continuous United States (U.S. Fish and Wildlife Service 2023) and as a special concern in Canada (COSEWIC 2014). In Europe, two wolverine populations have been defined: the Scandinavian and the Karelian population, and the species is listed as vulnerable (Andrén 2018). Consistent with global trends, previous studies highlight the need for ensuring gene flow between the populations in Finland and between other Eurasian populations (Lansink et al. 2020; Lansink et al. 2022).

The increase in the size of the Finnish wolverine population, and subsequent range expansion, could be attributed to protection measures and the availability of suitable denning habitats and prey. A similar range expansion towards boreal forests has been documented in the wolverine population in Sweden (Aronsson and Persson 2017). Nevertheless, as wolverines recolonize their historical range in Fennoscandia, they are likely to encounter challenges, particularly with increasing human activity towards the southern latitudes, and the extensive predator control in Norway, which could lead to a source‐sink population dynamic, especially in northern Finland (Gervasi et al. 2015, Fisher et al. 2022). Our findings show that in boreal forests wolverine occupancy would be promoted by mixed woodlands combining broadleaved trees with conifers and ensuring logging practices maintain habitat continuity between forest patches, especially in areas with increasing human development pressure. Alternatives to snow‐dependent monitoring programs should be considered, as decreasing snow depth and the expansion of wolverines into new, snow‐scarce areas in southern Finland may complicate future assessments of the Finnish wolverine population.

Our findings offer an overview of changes in wolverine occurrence in Finland. Consideration of factors known to affect wolverine habitat selection, such as interspecific competition (Koskela et al. 2013a; Rauset et al. 2013) and housing density (Balkenhol et al. 2020), could further explain the occurrence. Moreover, wolverines' dispersal patterns are affected by resource and mate competition (Vangen et al. 2001) and by cumulative effects of environmental change such as anthropogenic landscape change (Heim et al. 2017). While the MS‐NFI data provides a general understanding of wolverine occurrence, it lacks finer details, such as understory structure. Wildlife and field triangle data collection is a form of citizen science, which can present challenges with data accuracy (Balázs et al. 2021). However, with the large number of surveyed censuses, consistent counting methods, and careful data exploration, potential biases are reduced. Moreover, wolverines have a quite distinct paw print and pattern of tracks compared to other large mammals and much larger paw prints than other mustelids, and thus their tracks are relatively easy to identify.

To summarize, we showed that the forest and landscape structures that best explain wolverine occurrence were landscape fragmentation, tree volume, forest tree species composition, and distance to clearcuts. Areas were more likely to be occupied if forests were less fragmented and dominated by broadleaved trees, while the probability decreased if the total volume of trees was high or clearcuts were in close proximity. The significant structures explaining wolverine occurrence differed between spatial scales. The landscape scale was found to be more relevant when studying the impact of overall forest structure on occurrence. Future research could achieve a more detailed understanding of the occurrence of mustelids by incorporating for example, airborne laser scanning datasets, to better capture forest structure. Additionally, future studies should investigate how these forest and landscape structure preferences interact with other factors, such as prey availability, competition with other predators, human disturbance, and poaching mortality.

## Author Contributions

**Pinja‐Emilia Lämsä:** conceptualization (equal), formal analysis (lead), investigation (lead), methodology (equal), visualization (lead), writing – original draft (lead), writing – review and editing (lead). **Audrey Mercier:** conceptualization (equal), methodology (equal), writing – review and editing (supporting). **Andreas Lindén:** conceptualization (equal), data curation (lead), methodology (equal), writing – review and editing (supporting). **Aarne Hovi:** conceptualization (equal), methodology (equal), writing – review and editing (supporting). **Miina Rautiainen:** conceptualization (equal), methodology (equal), supervision (lead), writing – review and editing (supporting).

## Conflicts of Interest

The authors declare no conflicts of interest.

## Data Availability

Due to sensitivity of an endangered species, the wolverine snow‐track data are not shared. Other data that support the findings of this study are openly available in Luke MS‐NFI Download Service at https://kartta.luke.fi/index‐en.html, Finnish Transport Infrastructure Agency's repository at https://vayla.fi/en/transport‐network/data/digiroad/data, Finnish Environment Institute repository at https://www.syke.fi/en‐us/Open_information/Spatial_datasets/Downloadable_spatial_dataset, and National Land Survey of Finland’ repository at https://www.maanmittauslaitos.fi/en/maps‐and‐spatial‐data/datasets‐and‐interfaces/product‐descriptions/topographic‐database. The relevant scripts can be accessed on GitHub at https://github.com/pelamsa/wolverine_colonization.

## Appendix A

**TABLE A1:** Original set of covariates tested for multicollinearity. Each covariate was assessed for multicollinearity to ensure independence among predictors. Following VIF analysis, covariates were selected for the modeling based on low VIF value (VIF < 3) and theoretical relevance. CORINE Land Cover data are from Syke (partly LUKE, MAVI, LIVI, DVV, EU, MML Terrain Database 01/2017) and vector data sources are Syke, EEA, EU/Copernicus.

Predictor	Statistical techniques	Data source
Percentages of different site main classes in triangle	Percentage of class within a circular window	MS‐NFI “site main class” rasters 2011 and 2021
Stand age of trees	Average within a circular window	MS‐NFI 2011, 2021
Stem diameter at 1.3 m height	Average within a circular window	MS‐NFI 2011, 2021
Stand mean height of trees	Average within a circular window	MS‐NFI 2011, 2021
Percentage of broadleaved trees	Average within a circular window	MS‐NFI 2011, 2021, calculated using volume of different three species and total volume
Percentage of pine	Average within a circular window	MS‐NFI 2011, 2021, calculated using volume of different three species and total volume
Percentage of spruce	Average within a circular window	MS‐NFI 2011, 2021, calculated using volume of different three species and total volume
Canopy cover	Average within a circular window	MS‐NFI 2011, 2021
Stand basal area	Average within a circular window	MS‐NFI 2011, 2021
Volume, spruce	Average within a circular window	MS‐NFI 2011, 2021
Volume, pine	Average within a circular window	MS‐NFI 2011, 2021
Volume, broadleaved	Average within a circular window	MS‐NFI 2011, 2021
Volume, total	Average within a circular window	MS‐NFI 2011, 2021
**Distance to**		
Anthropogenic landscapes	Average distance to the variable (in meters) within a circular window	Corine Land Cover 2011, Corine Land Cover 2018, layers “continuous urban fabric” and “discontinuous urban fabric” combined with layers “non‐irrigated arable land” and “complex cultivation patterns”.
Roads	Average distance to the variable (in meters) within a circular window	Digiroad data (2014, 2021), Finnish Transportation Agency
Clearcuts	Average distance to the variable (in meters) within a circular window	Average distance to clearcuts in the second period. Clearcut was defined as the MS‐NFI (2011, 2021) “*forest land*” pixels that do not fulfill the forest definition (canopy cover > 10%, tree height > 5 m) and were subtracted from unfiltered “*forest land*” raster.
Water bodies	Average distance to the variable (in meters) within a circular window	Topographic database (3/2024), National Land Survey of Finland
**Forest fragmentation**		
Patch density	Number of forest patches per 100 ha	Forest fragmentation metric calculated from MS‐NFI (2011, 2021) “*forest land*” raster filtered based on forest definition (> 10% canopy cover and > 5 m tree height).
Edge density	The sum of all edges of forest in relation to the landscape area (meters per hectare)	Forest fragmentation metric calculated from MS‐NFI (2011, 2021) “*forest land*” raster filtered based on forest definition (> 10% canopy cover and > 5 m tree height).

**TABLE A2:** Variance inflation factor (VIF) values for preselected environmental covariates at two spatial scales: Local and landscape.

	Distance to clearcuts, 1st period	Distance to clearcuts, 2nd period	Patch density	Volume of pine	Total volume of trees	Proportion of broadleaved trees	Effort
Local scale	1.274	1.361	1.138	1.966	1.803	1.836	1.014
Landscape scale	2.093	2.375	1.099	1.807	2.031	1.787	1.004

**TABLE A3:** Model selection results at the local scale based on AIC_c_ values. Each candidate model is presented along with its number of parameters (k), AIC_c_ value, ΔAIC_c_ (the difference in AICc from the most parsimonious model), and Akaike weight (w). Models are ranked by their relative support, with lower AIC_c_ values indicating better model fit.

Model	k	AIC_c_	∆AIC_c_	w
PatchD + Broadleaved	5	595.782	0.000	0.147
Clearcut2 + PatchD + Broadleaved	6	596.423	0.641	0.107
PatchD + VolTotal + Broadleaved	6	597.093	1.311	0.077
Clearcut1 + Clearcut2 + PatchD + Broadleaved	7	597.257	1.474	0.071
Clearcut2 + PatchD + VolTotal + Broadleaved	7	597.397	1.615	0.066
Clearcut1 + PatchD + Broadleaved	6	597.426	1.644	0.065
PatchD + VolPine + VolTotal + Broadleaved	7	597.462	1.680	0.064
PatchD + VolPine + Broadleaved	6	597.463	1.681	0.064
Clearcut1 + Clearcut2 + PatchD + VolTotal + Broadleaved	8	597.490	1.708	0.063
Clearcut2 + PatchD + VolPine + VolTotal + Broadleaved	8	598.072	2.290	0.047
Clearcut2 + PatchD + VolPine + Broadleaved	7	598.284	2.502	0.042
Clearcut1 + PatchD + VolTotal + Broadleaved	7	598.473	2.691	0.038
Clearcut1 + Clearcut2 + PatchD + VolPine + VolTotal + Broadleaved	9	598.662	2.880	0.035
Clearcut1 + PatchD + VolPine + VolTotal + Broadleaved	8	599.097	3.314	0.028
Clearcut1 + PatchD + VolPine + Broadleaved	7	599.244	3.462	0.026
Clearcut1 + Clearcut2 + PatchD + VolPine + Broadleaved	8	599.280	3.498	0.026
Clearcut1 + Clearcut2 + PatchD	6	603.149	7.367	0.004
Clearcut1 + Clearcut2 + PatchD + VolPine	7	603.541	7.759	0.003
Clearcut2 + Broadleaved	5	604.083	8.301	0.002
Clearcut2 + PatchD	5	604.135	8.353	0.002
PatchD	4	604.249	8.467	0.002
Clearcut2 + VolTotal + Broadleaved	6	604.355	8.573	0.002
Clearcut1 + Clearcut2 + VolTotal + Broadleaved	7	604.735	8.953	0.002
Clearcut1 + Clearcut2 + PatchD + VolTotal	7	604.884	9.102	0.002
Clearcut2 + VolPine + VolTotal + Broadleaved	7	604.918	9.136	0.002
Clearcut1 + PatchD	5	604.958	9.176	0.001
Clearcut2 + PatchD + VolPine	6	605.201	9.419	0.001
Clearcut1 + Clearcut2 + Broadleaved	6	605.336	9.554	0.001
Clearcut1 + Clearcut2 + PatchD + VolPine + VolTotal	8	605.552	9.770	0.001
PatchD + VolPine	5	605.668	9.886	0.001
Clearcut1 + Clearcut2 + VolPine + VolTotal + Broadleaved	8	605.818	10.036	0.001
Clearcut2 + VolPine + Broadleaved	6	606.023	10.241	0.001
Clearcut1 + PatchD + VolPine	6	606.078	10.296	0.001
Clearcut2 + PatchD + VolTotal	6	606.163	10.381	0.001
PatchD + VolTotal	5	606.194	10.412	0.001
Clearcut1 + PatchD + VolTotal	6	606.985	11.203	0.001
Clearcut2 + PatchD + VolPine + VolTotal	7	607.106	11.324	0.001
Broadleaved	4	607.185	11.403	< 0.001
PatchD + VolPine + VolTotal	6	607.322	11.540	< 0.001
Clearcut1 + Clearcut2 + VolPine + Broadleaved	7	607.367	11.585	< 0.001
Clearcut1 + PatchD + VolPine + VolTotal	7	607.981	12.199	< 0.001
VolPine + VolTotal + Broadleaved	6	607.992	12.210	< 0.001
VolTotal + Broadleaved	5	608.189	12.407	< 0.001
VolPine + Broadleaved	5	608.779	12.997	< 0.001
Clearcut1 + Broadleaved	5	609.203	13.421	< 0.001
Clearcut1 + Clearcut2	5	609.618	13.836	< 0.001
Clearcut2	4	609.673	13.890	< 0.001
Clearcut1 + VolPine + VolTotal + Broadleaved	7	610.014	14.232	< 0.001
Clearcut1 + VolTotal + Broadleaved	6	610.113	14.331	< 0.001
Clearcut1 + Clearcut2 + VolPine	6	610.171	14.389	< 0.001
Clearcut1 + VolPine + Broadleaved	6	610.796	15.014	< 0.001
Clearcut2 + VolPine	5	610.842	15.060	< 0.001
Clearcut1 + Clearcut2 + VolTotal	6	610.942	15.160	< 0.001
Clearcut2 + VolTotal	5	611.590	15.808	< 0.001
Clearcut1 + Clearcut2 + VolPine + VolTotal	7	611.944	16.162	< 0.001
Clearcut2 + VolPine + VolTotal	6	612.869	17.087	< 0.001
Null	3	613.439	17.657	< 0.001
VolPine	4	615.181	19.399	< 0.001
Clearcut1	4	615.202	19.420	< 0.001
VolTotal	4	615.444	19.662	< 0.001
Clearcut1 + VolPine	5	616.823	21.041	< 0.001
VolPine + VolTotal	5	617.092	21.310	< 0.001
Clearcut1 + VolTotal	5	617.224	21.442	< 0.001
Clearcut1 + VolPine + VolTotal	6	618.810	23.028	< 0.001

**TABLE A4:** Model selection results at the landscape scale based on AIC_c_ values. Each candidate model is presented along with its number of parameters (*k*), AIC_c_ value, ΔAIC_c_ (the difference in AICc from the most parsimonious model), and Akaike weight (*w*). Models are ranked by their relative support, with lower AIC_c_ values indicating better model fit.

Model	*k*	AIC_c_	∆AIC_c_	*w*
Clearcut1 + Clearcut2 + PatchD + VolTotal + Broadleaved	8	581.250	0.000	0.587
Clearcut1 + Clearcut2 + PatchD + VolPine + VolTotal + Broadleaved	9	582.395	1.144	0.331
Clearcut1 + Clearcut2 + PatchD + Broadleaved	7	587.529	6.279	0.025
Clearcut1 + PatchD + VolPine + VolTotal + Broadleaved	8	588.735	7.485	0.014
Clearcut1 + Clearcut2 + PatchD + VolPine + Broadleaved	8	589.245	7.995	0.011
PatchD + VolPine + VolTotal + Broadleaved	7	590.713	9.463	0.005
Clearcut1 + PatchD + VolTotal + Broadleaved	7	590.728	9.477	0.005
Clearcut1 + PatchD + Broadleaved	6	590.976	9.726	0.005
Clearcut2 + PatchD + VolPine + VolTotal + Broadleaved	8	591.297	10.047	0.004
Clearcut1 + PatchD + VolPine + Broadleaved	7	592.550	11.300	0.002
PatchD + VolTotal + Broadleaved	6	592.903	11.653	0.002
PatchD + Broadleaved	5	593.033	11.783	0.002
Clearcut1 + Clearcut2 + VolTotal + Broadleaved	7	593.136	11.886	0.002
Clearcut2 + PatchD + Broadleaved	6	593.625	12.374	0.001
Clearcut2 + PatchD + VolTotal + Broadleaved	7	593.882	12.632	0.001
PatchD + VolPine + Broadleaved	6	594.497	13.247	0.001
Clearcut1 + Clearcut2 + PatchD + VolTotal	7	594.693	13.443	0.001
Clearcut1 + Clearcut2 + VolPine + VolTotal + Broadleaved	8	594.755	13.504	0.001
Clearcut2 + PatchD + VolPine + Broadleaved	7	594.813	13.563	0.001
Clearcut1 + Clearcut2 + PatchD + VolPine	7	595.151	13.901	0.001
Clearcut1 + Clearcut2 + PatchD + VolPine + VolTotal	8	595.250	14.000	0.001
Clearcut1 + Clearcut2 + PatchD	6	596.175	14.925	< 0.001
Clearcut1 + Clearcut2 + Broadleaved	6	601.959	20.709	< 0.001
Clearcut1 + Clearcut2 + VolTotal	6	602.003	20.753	< 0.001
Clearcut1 + Clearcut2 + VolPine + VolTotal	7	602.690	21.440	< 0.001
Clearcut1 + Clearcut2 + VolPine + Broadleaved	7	603.127	21.877	< 0.001
VolPine + VolTotal + Broadleaved	6	603.925	22.675	< 0.001
PatchD	4	604.060	22.810	< 0.001
Clearcut1 + VolPine + VolTotal + Broadleaved	7	604.146	22.895	< 0.001
Clearcut1 + Clearcut2 + VolPine	6	604.485	23.234	< 0.001
Clearcut1 + PatchD	5	605.314	24.064	< 0.001
Clearcut1 + Clearcut2	5	605.650	24.400	< 0.001
Clearcut2 + VolPine + VolTotal + Broadleaved	7	605.822	24.572	< 0.001
Clearcut2 + PatchD	5	606.033	24.783	< 0.001
Clearcut1 + VolTotal + Broadleaved	6	606.054	24.804	< 0.001
PatchD + VolTotal	5	606.073	24.822	< 0.001
PatchD + VolPine	5	606.082	24.832	< 0.001
VolTotal + Broadleaved	5	606.277	25.027	< 0.001
Broadleaved	4	606.782	25.532	< 0.001
Clearcut1 + Broadleaved	5	607.014	25.764	< 0.001
Clearcut1 + PatchD + VolTotal	6	607.315	26.065	< 0.001
Clearcut1 + PatchD + VolPine	6	607.335	26.084	< 0.001
Clearcut2 + PatchD + VolTotal	6	608.058	26.808	< 0.001
Clearcut2 + PatchD + VolPine	6	608.060	26.810	< 0.001
PatchD + VolPine + VolTotal	6	608.099	26.849	< 0.001
Clearcut2 + VolTotal + Broadleaved	6	608.264	27.014	< 0.001
VolPine + Broadleaved	5	608.309	27.058	< 0.001
Clearcut2 + Broadleaved	5	608.665	27.415	< 0.001
Clearcut1 + VolPine + Broadleaved	6	608.718	27.468	< 0.001
Clearcut1 + PatchD + VolPine + VolTotal	7	609.314	28.064	< 0.001
Clearcut2 + PatchD + VolPine + VolTotal	7	610.090	28.840	< 0.001
Clearcut2 + VolPine + Broadleaved	6	610.120	28.870	< 0.001
Null	3	613.439	32.189	< 0.001
Clearcut2	4	615.000	33.750	< 0.001
Clearcut1	4	615.022	33.772	< 0.001
VolPine	4	615.441	34.191	< 0.001
VolTotal	4	615.444	34.193	< 0.001
Clearcut2 + VolTotal	5	616.934	35.684	< 0.001
Clearcut2 + VolPine	5	617.014	35.764	< 0.001
Clearcut1 + VolTotal	5	617.022	35.772	< 0.001
Clearcut1 + VolPine	5	617.045	35.794	< 0.001
VolPine + VolTotal	5	617.423	36.173	< 0.001
Clearcut2 + VolPine + VolTotal	6	618.889	37.639	< 0.001
Clearcut1 + VolPine + VolTotal	6	619.043	37.792	< 0.001

## References

[ece371300-bib-0001] Allouche, O. , A. Tsoar , and R. Kadmon . 2006. “Assessing the Accuracy of Species Distribution Models: Prevalence, Kappa and the True Skill Statistic (TSS).” Journal of Applied Ecology 43, no. 6: 1223–1232. 10.1111/j.1365-2664.2006.01214.x.

[ece371300-bib-0002] Andrén, H. 2018. “*Gulo gulo* (Europe Assessment) (Errata Version Published in 2019).” IUCN Red List of Threatened Species, 2018(e.T9561A144336120). Accessed April 8, 2024. https://www.iucnredlist.org/en.

[ece371300-bib-0003] Arnold, T. W. 2010. “Uninformative Parameters and Model Selection Using Akaike's Information Criterion.” Journal of Wildlife Management 74, no. 6: 1175–1178. 10.1111/j.1937-2817.2010.tb01236.x.

[ece371300-bib-0005] Aronsson, M. , and J. Persson . 2017. “Mismatch Between Goals and the Scale of Actions Constrains Adaptive Carnivore Management: The Case of the Wolverine in Sweden.” Animal Conservation 20, no. 3: 261–269. 10.1111/acv.12310.

[ece371300-bib-0004] Aronsson, M. , H. Andrén , M. Low , and J. Persson . 2023. “Wolverine Denning Behaviour and Its Implications for Monitoring Reproductive Females.” Wildlife Biology 2023, no. 4: e01079. 10.1002/wlb3.01079.

[ece371300-bib-0006] Baguette, M. , S. Blanchet , D. Legrand , V. M. Stevens , and C. Turlure . 2013. “Individual Dispersal, Landscape Connectivity and Ecological Networks.” Biological Reviews 88, no. 2: 310–326. 10.1111/brv.12000. 23176626

[ece371300-bib-0007] Balázs, B. , P. Mooney , E. Nováková , L. Bastin , and J. Jokar Arsanjani . 2021. “Data Quality in Citizen Science.” In The Science of Citizen Science, edited by K. Vohland , A. Land‐Zandstra , L. Ceccaroni , et al., 139–157. Springer International Publishing. 10.1007/978-3-030-58278-4_8.

[ece371300-bib-0008] Balkenhol, N. , M. K. Schwartz , R. M. Inman , et al. 2020. “Landscape Genetics of Wolverines ( *Gulo gulo* ): Scale‐Dependent Effects of Bioclimatic, Topographic, and Anthropogenic Variables.” Journal of Mammalogy 101, no. 3: 790–803. 10.1093/jmammal/gyaa037. 32665742 PMC7333878

[ece371300-bib-0009] Barrueto, M. , A. Forshner , J. Whittington , A. P. Clevenger , and M. Musiani . 2022. “Protection Status, Human Disturbance, Snow Cover and Trapping Drive Density of a Declining Wolverine Population in the Canadian Rocky Mountains.” Scientific Reports 12, no. 1: 17412. 10.1038/s41598-022-21499-4. 36280695 PMC9592595

[ece371300-bib-0010] Barsugli, J. J. , A. J. Ray , B. Livneh , et al. 2020. “Projections of Mountain Snowpack Loss for Wolverine Denning Elevations in the Rocky Mountains.” Earth's Future 8, no. 10: e2020EF001537. 10.1029/2020EF001537.

[ece371300-bib-0011] Bivand, R. 2022. “R Packages for Analyzing Spatial Data: A Comparative Case Study With Areal Data.” Geographical Analysis 54, no. 3: 488–518. 10.1111/gean.12319.

[ece371300-bib-0012] Brooks, M. , B. Bolker , K. Kristensen , et al. 2017. “glmmTMB Balances Speed and Flexibility Among Packages for Zero‐Inflated Generalized Linear Mixed Modeling (Version 1.1.10) [Computer Software].” 10.32614/CRAN.package.glmmTMB.

[ece371300-bib-0013] Bujnáková, D. , G. M. J. Lansink , A. V. Abramov , et al. 2024. “Expanding From Local to Continental Scale—A Genetic Assessment of the Eurasian Wolverine.” Diversity and Distributions 30, no. 7: e13846. 10.1111/ddi.13846.

[ece371300-bib-0014] Burnham, K. P. , and D. R. Anderson . 2002. Model Selection and Multimodel Inference. Vol. 488. Springer. 10.1007/b97636.

[ece371300-bib-0015] Carroll, K. A. , A. J. Hansen , R. M. Inman , and R. L. Lawrence . 2021. “Evaluating the Importance of Wolverine Habitat Predictors Using a Machine Learning Method.” Journal of Mammalogy 102, no. 6: 1466–1472. 10.1093/jmammal/gyab088.

[ece371300-bib-0016] Castagneyrol, B. , and H. Jactel . 2012. “Unraveling Plant–Animal Diversity Relationships: A Meta‐Regression Analysis.” Ecology 93, no. 9: 2115–2124. 10.1890/11-1300.1. 23094383

[ece371300-bib-0017] Copeland, J. P. , K. S. McKelvey , K. B. Aubry , et al. 2010. “The Bioclimatic Envelope of the Wolverine ( *Gulo gulo* ): Do Climatic Constraints Limit Its Geographic Distribution?” Canadian Journal of Zoology 88, no. 233–246: 233–246. 10.1139/Z09-136.

[ece371300-bib-0018] COSEWIC . 2014. COSEWIC Assessment and Status Report on the Wolverine Gulo Gulo in Canada, xi+76. Committee on the Status of Endangered Wildlife in Canada.

[ece371300-bib-0019] Davies, A. B. , and G. P. Asner . 2014. “Advances in Animal Ecology From 3D‐LiDAR Ecosystem Mapping.” Trends in Ecology & Evolution 29, no. 12: 681–691. 10.1016/j.tree.2014.10.005. 25457158

[ece371300-bib-0081] Day, C. C. , E. L. Landguth , M. A. Sawaya , et al. 2024. “Genetic Connectivity of Wolverines in Western North America.” Scientific Reports 14, no. 1. 10.1038/s41598-024-77956-9. PMC11568290 39548133

[ece371300-bib-0020] Fenlon, C. , L. O'Grady , M. L. Doherty , and J. Dunnion . 2018. “A Discussion of Calibration Techniques for Evaluating Binary and Categorical Predictive Models.” Preventive Veterinary Medicine 149: 107–114. 10.1016/j.prevetmed.2017.11.018. 29290291

[ece371300-bib-0021] Fisher, J. T. , S. Bradbury , B. Anholt , et al. 2013. “Wolverines ( *Gulo gulo luscus* ) on the Rocky Mountain Slopes: Natural Heterogeneity and Landscape Alteration as Predictors of Distribution.” Canadian Journal of Zoology 91, no. 10: 706–716. 10.1139/cjz-2013-0022.

[ece371300-bib-0022] Fisher, J. T. , S. Murray , M. Barrueto , et al. 2022. “Wolverines ( *Gulo gulo* ) in a Changing Landscape and Warming Climate: A Decadal Synthesis of Global Conservation Ecology Research.” Global Ecology and Conservation 34: e02019. 10.1016/j.gecco.2022.e02019.

[ece371300-bib-0023] Freeman, E. , and G. Moisen . 2008. “PresenceAbsence: An R Package for Presence Absence Analysis (Version 1.1.11) [Computer Software].” 10.18637/jss.v023.i11.

[ece371300-bib-0080] Gervasi, V. , H. Brøseth , E. B. Nilsen , H. Ellegren , Ø. Flagstad , and J. D. C. Linnell . 2015. “Compensatory Immigration Counteracts Contrasting Conservation Strategies of Wolverines (Gulo gulo) within Scandinavia.” Biological Conservation 191: 632–639. 10.1016/j.biocon.2015.07.024.

[ece371300-bib-0024] Gough, M. C. , and S. P. Rushton . 2000. “The Application of GIS‐Modelling to Mustelid Landscape Ecology.” Mammal Review 30, no. 3–4: 197–216. 10.1046/j.1365-2907.2000.00067.x.

[ece371300-bib-0025] Haddad, N. M. , L. A. Brudvig , J. Clobert , et al. 2015. “Habitat Fragmentation and Its Lasting Impact on Earth's Ecosystems.” Science Advances 1, no. 2: e1500052. 10.1126/sciadv.1500052. 26601154 PMC4643828

[ece371300-bib-0026] Hartig, F. , L. Lohse , and M. d. S. leite . 2022. “DHARMa: Residual Diagnostics for Hierarchical (Multi‐Level/Mixed) Regression Models (Version 0.4.7) [Computer Software].” 10.32614/CRAN.package.DHARMa.

[ece371300-bib-0027] Hayne, D. W. 1949. “Calculation of Size of Home Range1.” Journal of Mammalogy 30, no. 1: 1–18. 10.2307/1375189.

[ece371300-bib-0028] Heim, N. , J. T. Fisher , A. Clevenger , J. Paczkowski , and J. Volpe . 2017. “Cumulative Effects of Climate and Landscape Change Drive Spatial Distribution of Rocky Mountain Wolverine ( *Gulo gulo* L.).” Ecology and Evolution 7, no. 21: 8903–8914. 10.1002/ece3.3337. 29152186 PMC5677488

[ece371300-bib-0029] Hesselbarth, M. H. K. , M. Sciaini , K. A. With , K. Wiegand , and J. Nowosad . 2019. “Landscapemetrics: An Open‐Source R Tool to Calculate Landscape Metrics.” Ecography 42: 1648–1657. 10.32614/CRAN.package.landscapemetrics.

[ece371300-bib-0030] Hijmans, R. J. 2024. “terra: Spatial Data Analysis (Version 1.7–74) [Computer Software].” 10.32614/CRAN.package.terra.

[ece371300-bib-0031] Hiltunen, M. , K. Kauhala , and H. Lindén . 2004. “Habitat Use of the Mountain Hare *Lepus Timidus* in Summer: The Importance of Different Vegetation Layers.” Acta Theriologica 49, no. 4: 479–490. 10.1007/BF03192592.

[ece371300-bib-0032] Hosmer, D. W. , S. Lemeshow , and R. X. Sturdivant . 2013. Applied Logistic Regression. 1st ed. Wiley. 10.1002/9781118548387.

[ece371300-bib-0033] Hyvärinen, E. , A. Juslén , E. Kemppainen , A. Uddström , and U.‐M. Liukko . 2019. Suomen Lajien uhanalaisuus: Punainen Kirja 2019. Ympäristöministeriö & Suomen ympäristökeskus. http://hdl.handle.net/10138/299501.

[ece371300-bib-0034] Isbell, F. , P. Balvanera , A. S. Mori , et al. 2023. “Expert Perspectives on Global Biodiversity Loss and Its Drivers and Impacts on People.” Frontiers in Ecology and the Environment 21, no. 2: 94–103. 10.1002/fee.2536.

[ece371300-bib-0035] Jackson, H. B. , and L. Fahrig . 2015. “Are Ecologists Conducting Research at the Optimal Scale?” Global Ecology and Biogeography 24, no. 1: 52–63. 10.1111/geb.12233.

[ece371300-bib-0036] Jokinen, M. E. , S. M. Webb , D. L. Manzer , and R. B. Anderson . 2019. “Characteristics of Wolverine (*Gulo Gulo*) Dens in the Lowland Boreal Forest of North‐Central Alberta.” Canadian Field‐Naturalist 133: 1–15. 10.22621/cfn.v133i1.2083.

[ece371300-bib-0037] Kohl, M. 2023. “MKclass: Statistical Classification (Version 0.5) [Computer Software].” 10.32614/CRAN.package.MKclass.

[ece371300-bib-0038] Kojola, I. , S. Heikkinen , M. Samu , and T. Ollila . 2023. “Ahmakanta Suomessa 2023.” Luonnonvara‐ Ja Biotalouden Tutkimus 123, no. 2023: 11.

[ece371300-bib-0039] Korhonen, K. T. , A. Ahola , J. Heikkinen , et al. 2021. “Forests of Finland 2014–2018 and Their Development 1921–2018.” Silva Fennica 55, no. 5. 10.14214/sf.10662.

[ece371300-bib-0040] Korhonen, K. T. , M. Räty , H. Haakana , et al. 2024. “Forests of Finland 2019–2023 and Their Development 1921–2023.” Silva Fennica 58, no. 5. https://www.silvafennica.fi/article/24045.

[ece371300-bib-0042] Koskela, A. , I. Kojola , J. Aspi , and M. Hyvarinen . 2013b. “The Diet of Breeding Female Wolverines ( *Gulo gulo* ) in Two Areas of Finland.” Acta Theriologica 58: 199–204. 10.1007/s13364-012-0111-z.

[ece371300-bib-0041] Koskela, A. , S. Kaartinen , J. Aspi , I. Kojola , P. Helle , and S. Rytkönen . 2013a. “Does Grey Wolf Presence Affect Habitat Selection of Wolverines?” Annales Zoologici Fennici 50, no. 4: 216–224. 10.5735/085.050.0405.

[ece371300-bib-0043] Kuhn, M. 2008. “Building Predictive Models in R Using the Caret Package.” Journal of Statistical Software 28, no. 5: 1–26. 10.18637/jss.v028.i05.27774042

[ece371300-bib-0044] Kulju, I. , T. Niinistö , A. Peltola , et al. 2023. “Metsätilastollinen vuosikirja 2022.” Luonnonvarakeskus. https://jukuri.luke.fi/handle/10024/553167.

[ece371300-bib-0046] Lansink, G. M. J. , O. Kleven , R. Ekblom , et al. 2022. “Potential for Increased Connectivity Between Differentiated Wolverine Populations.” Biological Conservation 272: 109601. 10.1016/j.biocon.2022.109601.

[ece371300-bib-0045] Lansink, G. M. J. , R. Esparza‐Salas , M. Joensuu , et al. 2020. “Population Genetics of the Wolverine in Finland: The Road to Recovery?” Conservation Genetics 21, no. 3: 481–499. 10.1007/s10592-020-01264-8.

[ece371300-bib-0047] Larivière, S. , and A. P. Jennings . 2009. “Family Mustelidae (Weasels and Relatives).” In Handbook of the Mammals of the World, edited by D. E. Wilson and R. A. Mittermeier , vol. 1, 564–656. Carnivores. Lynx Edicions.

[ece371300-bib-0048] Lindén, H. , E. Helle , P. Helle , and M. Wikman . 1996. “Wildlife Triangle Scheme in Finland: Methods and Aims for Monitoring Wildlife Populations.” Finnish Game Research 49: 4–11.

[ece371300-bib-0049] Lofroth, E. C. , and J. Krebs . 2007. “The Abundance and Distribution of Wolverines in British Columbia, Canada.” Journal of Wildlife Management 71, no. 7: 2159–2169. 10.2193/2007-094.

[ece371300-bib-0050] LUKE . 2024. “The Natural Resources Institute Finland Guidelines for Field Triangle Censuses.” Accessed March 7, 2024. https://oma.riistakolmiot.fi/ohje/peltokolmiot.

[ece371300-bib-0051] Luomaranta, A. , J. Aalto , and K. Jylhä . 2019. “Snow Cover Trends in Finland Over 1961–2014 Based on Gridded Snow Depth Observations.” International Journal of Climatology 39, no. 7: 3147–3159. 10.1002/joc.6007.

[ece371300-bib-0052] Macdonald, D. W. , L. A. Harrington , and C. Newman . 2017. “Dramatis Personae: An Introduction to the Wild Musteloids.” In Biology and Conservation of Musteloids, edited by D. W. Macdonald , C. Newman , and L. A , 3–74. Oxford University Press. 10.1093/oso/9780198759805.001.0001.

[ece371300-bib-0053] Malcangi, F. , A. Lindén , J. Sundell , and J. Loehr . 2024. “Correlation Between Mammal Track Abundance and Forest Landscape Integrity Index Validates Actual Forest Ecological Integrity.” Oecologia 206: 61–72. 10.1007/s00442-024-05613-z. 39230725 PMC11489168

[ece371300-bib-0054] May, R. , L. Gorini , J. Van Dijk , H. Brøseth , J. D. C. Linnell , and A. Landa . 2012. “Habitat Characteristics Associated With Wolverine den Sites in Norwegian Multiple‐Use Landscapes.” Journal of Zoology 287, no. 3: 195–204. 10.1111/j.1469-7998.2012.00907.x.

[ece371300-bib-0055] Mazziotta, A. , A. Lindén , K. Eyvindson , et al. 2024. “Unraveling the Characteristic Spatial Scale of Habitat Selection for Forest Grouse Species in the Boreal Landscape.” Forest Ecology and Management 563: 122008. 10.1016/j.foreco.2024.122008.

[ece371300-bib-0056] McKelvey, K. S. , J. P. Copeland , M. K. Schwartz , et al. 2011. “Climate Change Predicted to Shift Wolverine Distributions, Connectivity, and Dispersal Corridors.” Ecological Applications 21, no. 8: 2882–2897. 10.1890/10-2206.1.

[ece371300-bib-0057] Melin, M. , P. Packalén , J. Matala , L. Mehtätalo , and J. Pusenius . 2013. “Assessing and Modeling Moose ( *Alces alces* ) Habitats With Airborne Laser Scanning Data.” International Journal of Applied Earth Observation and Geoinformation 23: 389–396. 10.1016/j.jag.2012.11.004.

[ece371300-bib-0058] Mönkkönen, M. , T. Aakala , C. Blattert , et al. 2022. “More Wood but Less Biodiversity in Forests in Finland: A Historical Evaluation.” Memoranda Societatis Pro Fauna et Flora Fennica 98, no. Supplement 2: 1–11. https://journal.fi/msff/article/view/120306.

[ece371300-bib-0059] Moqanaki, E. , C. Milleret , P. Dupont , H. Brøseth , and R. Bischof . 2023. “Wolverine Density Distribution Reflects Past Persecution and Current Management in Scandinavia.” Ecography 2023, no. 9: e06689. 10.1111/ecog.06689.

[ece371300-bib-0060] Persson, J. , P. Wedholm , and P. Segerström . 2010. “Space Use and Territoriality of Wolverines ( *Gulo gulo* ) in Northern Scandinavia.” European Journal of Wildlife Research 56, no. 1: 49–57. 10.1007/s10344-009-0290-3.

[ece371300-bib-0061] Planillo, A. , M. Wenzler‐Meya , I. Reinhardt , et al. 2024. “Understanding Habitat Selection of Range‐Expanding Populations of Large Carnivores: 20 Years of Grey Wolves ( *Canis lupus* ) Recolonizing Germany.” Diversity and Distributions 30, no. 1: 71–86. 10.1111/ddi.13789.

[ece371300-bib-0062] Poley, L. G. , A. J. Magoun , M. D. Robards , and R. L. Klimstra . 2018. “Distribution and Occupancy of Wolverines on Tundra, Northwestern Alaska.” Journal of Wildlife Management 82, no. 5: 991–1002. 10.1002/jwmg.21439.

[ece371300-bib-0063] R Core Team . 2024. R: A Language and Environment for Statistical Computing. R Foundation for Statistical Computing. https://www.R‐project.org.

[ece371300-bib-0064] Rauset, G. R. , J. Mattisson , H. Andrén , G. Chapron , and J. Persson . 2013. “When Species' Ranges Meet: Assessing Differences in Habitat Selection Between Sympatric Large Carnivores.” Oecologia 172, no. 3: 701–711. 10.1007/s00442-012-2546-y. 23242426

[ece371300-bib-0065] Salemaa, M. , J.‐P. Hotanen , J. Oksanen , T. Tonteri , and P. Merilä . 2023. “Broadleaved Trees Enhance Biodiversity of the Understorey Vegetation in Boreal Forests.” Forest Ecology and Management 546: 121357. 10.1016/j.foreco.2023.121357.

[ece371300-bib-0066] Savola, S. , H. Henttonen , and H. Lindén . 2013. “Vole Population Dynamics During the Succession of a Commercial Forest in Northern Finland.” Annales Zoologici Fennici 50, no. 1–2: 79–88. 10.5735/086.050.0107.

[ece371300-bib-0067] Sawaya, M. A. , A. P. Clevenger , and M. K. Schwartz . 2019. “Demographic Fragmentation of a Protected Wolverine Population Bisected by a Major Transportation Corridor.” Biological Conservation 236: 616–625. 10.1016/j.biocon.2019.06.030.

[ece371300-bib-0069] Scrafford, M. A. , J. L. Seguin , L. K. McCaw , M. S. Boyce , and J. C. Ray . 2024. “Wolverine Density, Survival, and Population Trends in the Canadian Boreal Forest.” Journal of Wildlife Management 88, no. 5: e22587. 10.1002/jwmg.22587.

[ece371300-bib-0068] Scrafford, M. A. , T. Avgar , B. Abercrombie , J. Tigner , and M. S. Boyce . 2017. “Wolverine Habitat Selection in Response to Anthropogenic Disturbance in the Western Canadian Boreal Forest.” Forest Ecology and Management 395: 27–36. 10.1016/j.foreco.2017.03.029.

[ece371300-bib-0070] Signorell, A. , K. Aho , A. Alfons , et al. 2024. “DescTools: Tools for Descriptive Statistics (Version 0.99.57) [Computer software].” 10.32614/CRAN.package.DescTools.

[ece371300-bib-0071] Stuber, E. F. , and J. J. Fontaine . 2019. “How Characteristic Is the Species Characteristic Selection Scale?” Global Ecology and Biogeography 28, no. 12: 1839–1854. 10.1111/geb.12998.

[ece371300-bib-0072] Thuiller, W. , S. Lavorel , M. T. Sykes , and M. B. Araújo . 2006. “Using Niche‐Based Modelling to Assess the Impact of Climate Change on Tree Functional Diversity in Europe.” Diversity and Distributions 12, no. 1: 49–60. 10.1111/j.1366-9516.2006.00216.x.

[ece371300-bib-0073] Tomppo, E. , M. Haakana , M. Katila , and J. Peräsaari . 2008. Multi‐Source National Forest Inventory. Methods and Applications. Vol. 18. Springer. 374. 10.1007/978-1-4020-8713-4.

[ece371300-bib-0074] Tomppo, E. , M. Katila , K. Mäkisara , and J. Peräsaari . 2014. “The Multi‐Source National Forest Inventory of Finland – Methods and Results 2011.” Working Papers of the Finnish Forest Research Institute 319. http://urn.fi/URN:ISBN:978‐951‐40‐2516‐7.

[ece371300-bib-0075] U.S. Fish and Wildlife Service . 2023. “Endangered and Threatened Wildlife and Plants; Threatened Species Status With Section 4(d) Rule for the Contiguous United States Distinct Population Segment of the North American Wolverine (*Gulo Gulo Luscus*).” Federal Register 88, no. 229: 73378–73413. https://www.federalregister.gov/documents/2023/11/30/2023‐26206/endangered‐and‐threatened‐wildlife‐and‐plants‐threatened‐species‐status‐with‐section‐4d‐rule‐for.

[ece371300-bib-0076] van der Veen, B. , J. Mattisson , B. Zimmermann , J. Odden , and J. Persson . 2020. “Refrigeration or Anti‐Theft? Food‐Caching Behavior of Wolverines ( *Gulo gulo* ) in Scandinavia.” Behavioral Ecology and Sociobiology 74, no. 5: 52. 10.1007/s00265-020-2823-4.

[ece371300-bib-0077] Vangen, K. M. , J. Persson , A. Landa , R. Andersen , and P. Segerström . 2001. “Characteristics of Dispersal in Wolverines.” Canadian Journal of Zoology 79, no. 9: 1641–1649. 10.1139/z01-124.

[ece371300-bib-0078] Zelnik, Y. R. , M. Barbier , D. W. Shanafelt , M. Loreau , and R. M. Germain . 2024. “Linking Intrinsic Scales of Ecological Processes to Characteristic Scales of Biodiversity and Functioning Patterns.” Oikos 2024, no. 3: e10514. 10.1111/oik.10514.

[ece371300-bib-0079] Zuur, A. F. , E. N. Ieno , and C. S. Elphick . 2010. “A Protocol for Data Exploration to Avoid Common Statistical Problems.” Methods in Ecology and Evolution 1, no. 1: 3–14. 10.1111/j.2041-210X.2009.00001.x.

